# 1-Cyano­methyl-1,4-diazo­niabicyclo­[2.2.2]octane tetra­chloridocobaltate(II)

**DOI:** 10.1107/S1600536812017187

**Published:** 2012-04-25

**Authors:** Yi Zhang, Bo-Han Zhu

**Affiliations:** a Ordered Matter Science Research Center, Southeast University, Nanjing 211189, People’s Republic of China

## Abstract

In the title salt, (C_8_H_15_N_3_)[CoCl_4_], the four chloride anions coordinate the Co^II^ ion in a distorted tetra­hedral geometry. In the crystal, N—H⋯Cl hydrogen bonds link cations and anions into chains running along the *c* axis. The crystal packing is further stabilized by weak C—H⋯Cl and C—H⋯N inter­actions.

## Related literature

Crystal structures of related Cu and Cd analogs were reported by Wei (2010 ▶) and Zhang & Zhu (2012 ▶), respectively. For ferroelectric properties of 1,4-diaza­bicyclo­[2.2.2]octane deriv­atives, see: Zhang *et al.* (2009 ▶, 2010 ▶). 
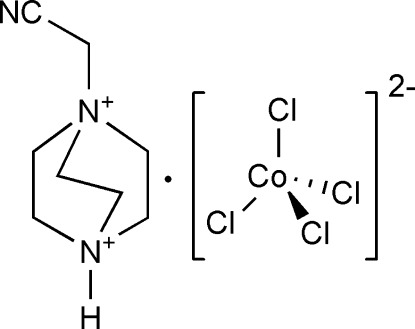


## Experimental

### 

#### Crystal data

(C_8_H_15_N_3_)[CoCl_4_]
*M* *_r_* = 353.96
Monoclinic, 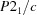

*a* = 8.3085 (17) Å
*b* = 13.604 (3) Å
*c* = 12.185 (2) Å
β = 93.78 (3)°
*V* = 1374.3 (5) Å^3^
*Z* = 4
Mo *K*α radiation
μ = 2.00 mm^−1^
*T* = 298 K
0.36 × 0.32 × 0.28 mm

#### Data collection

Rigaku Mercury70 CCD diffractometer
Absorption correction: multi-scan (*CrystalClear*; Rigaku, 2005 ▶) *T*_min_ = 0.491, *T*_max_ = 0.571
13757 measured reflections
3152 independent reflections
2724 reflections with *I* > 2σ(*I*)
*R*_int_ = 0.057

#### Refinement

*R*[*F* ^2^ > 2σ(*F* ^2^)] = 0.046
*wR*(*F* ^2^) = 0.135
*S* = 0.98
3152 reflections
149 parameters
H atoms treated by a mixture of independent and constrained refinement
Δρ_max_ = 0.58 e Å^−3^
Δρ_min_ = −0.52 e Å^−3^

### 

Data collection: *SCXmini Benchtop Crystallography System Software* (Rigaku, 2006 ▶); cell refinement: *SCXmini Benchtop Crystallography System Software*; data reduction: *SCXmini Benchtop Crystallography System Software*; program(s) used to solve structure: *SHELXS97* (Sheldrick, 2008 ▶); program(s) used to refine structure: *SHELXL97* (Sheldrick, 2008 ▶); molecular graphics: *SHELXTL* (Sheldrick, 2008 ▶); software used to prepare material for publication: *SHELXL97*.

## Supplementary Material

Crystal structure: contains datablock(s) I, global. DOI: 10.1107/S1600536812017187/cv5283sup1.cif

Structure factors: contains datablock(s) I. DOI: 10.1107/S1600536812017187/cv5283Isup2.hkl

Additional supplementary materials:  crystallographic information; 3D view; checkCIF report

## Figures and Tables

**Table 1:** Hydrogen-bond geometry (Å, °)

*D*—H⋯*A*	*D*—H	H⋯*A*	*D*⋯*A*	*D*—H⋯*A*
N2—H10⋯Cl3^i^	0.86 (5)	2.52 (5)	3.236 (3)	140 (4)
N2—H10⋯Cl2^ii^	0.86 (5)	2.65 (5)	3.225 (3)	125 (4)
C3—H3*B*⋯Cl1^iii^	0.97	2.74	3.647 (4)	156
C7—H7*A*⋯Cl2^iii^	0.97	2.58	3.492 (4)	156
C2—H2*A*⋯Cl3^iv^	0.97	2.73	3.543 (4)	142
C3—H3*A*⋯N3^v^	0.97	2.58	2.983 (4)	105

Symmetry codes: (i) 
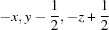
; (ii) 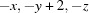; (iii) 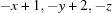; (iv) 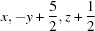; (v) 
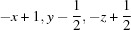
.

**Fig. 1.**
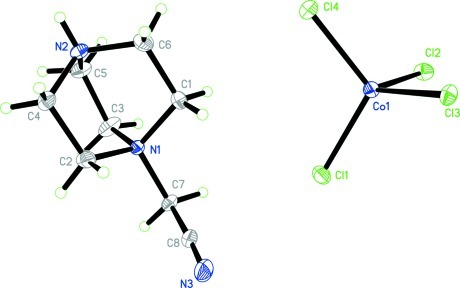
Molecular structure of the title compound, with the atomic numbering scheme. Displacement ellipsoids are drawn at the 30% probability level.

**Fig. 2.**
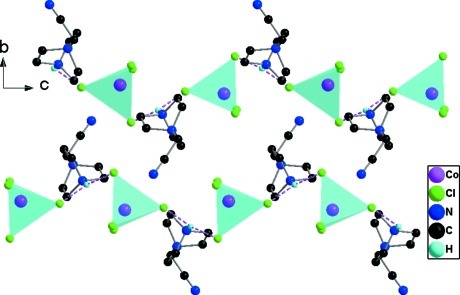
A portion of the crystal packing viewed along the a axis. Dotted lines indicate N—H···Cl hydrogen bonds.

## supplementary crystallographic information

### Comment

The title compound, (I), has been obtained in the framework of a systematic investigation of dielectric-ferroelectric materials containing 1,4-diazabicyclo[2.2.2]octane (DABCO) (Zhang, Ye *et al.*, 2009; Zhang, Ye *et al.*, 2010). The asymmetric unit of (I) (Fig. 1) contains one cation, (C_8_H_15_N_3_)^2+^, and one anion, (CoCl_4_)^2-^. All bond lengths and angles are normal and correspond to those observed in isostructural Cu (Wei, 2010) and Cd (Zhang & Zhu, 2012) analogs. The Co centers are coordinated by four Cl atoms with very similar distances in the range of2.2749 (12) to 2.2910 (12) Å. The Cl—Co—Cl bond angles are between 103.21 (4) and 113.85 (5) ° which shows that the coordination polyhedron can be described as a slightly distorted tetrahedron. The ammonium groups of the organic cations are engaged in bifurcated hydrogen bonds to chlorine atoms of two (CoCl_4_)^2-^ anions. These weak N—H···Cl interactions cause the formation of a one-dimensional chain along the [0 0 1] (Fig. 2). The crystal packing is further stabilized by the weak intermolecular C—H···Cl and C—H···N interactions (Table 2).

### Experimental

Chloroacetonitrile(0.1 mol, 7.55 g) was added to a CH_3_CN (25 ml) solution of 1,4-Diaza-bicyclo[2.2.2]octane (DABCO) (0.1 mol, 11.2 g) with stirring for 1 h at room temperature. 1-(cyanomethyl)-4-aza-1-azonia-bicyclo[2.2.2]octane chloride quickly formed as white solid was filtered, washed with acetonitrile and dried (yield: 80%). CoCl_2_.6H_2_O (0.01 mol, 2.38 g) and 1 g 36% HCl were dissolved in H_2_O (20 ml) and 1-(cyanomethyl)-4-aza-1-azonia-bicyclo[2.2.2]octane chloride (0.01 mol, 1.875 g) in H_2_O (20 ml) was added. The resulting solution was stirred until a clear solution was obtained. After slow evaporation of the solvent, blue block crystals of the title compound suitable for X-ray analysis were obtained in about 60% yield.The title compound has no dielectric disuniform from 80 K to 373 K, (m.p. > 373 K).

### Refinement

N-bound atom H1 was located on a difference map and isotropically refined. C-bound H atoms were geometrically positioned (C—H 0.97 Å) and refined as riding, with *U*_iso_(H) =1.2 *U*_eq_(C).

### Crystal data

**Table:**

(C_8_H_15_N_3_)[CoCl_4_]	*F*(000) = 716
*M**_r_* = 353.96	*D*_x_ = 1.711 Mg m^−^^3^
Monoclinic, *P*2_1_/*c*	Mo *K*α radiation, λ = 0.71073 Å
Hall symbol: -P 2ybc	Cell parameters from 2622 reflections
*a* = 8.3085 (17) Å	θ = 3.1–27.5°
*b* = 13.604 (3) Å	µ = 2.00 mm^−^^1^
*c* = 12.185 (2) Å	*T* = 298 K
β = 93.78 (3)°	Block, blue
*V* = 1374.3 (5) Å^3^	0.36 × 0.32 × 0.28 mm
*Z* = 4	

### Data collection

**Table:**

Rigaku Mercury70 CCD diffractometer	3152 independent reflections
Radiation source: fine-focus sealed tube	2724 reflections with *I* > 2σ(*I*)
Graphite monochromator	*R*_int_ = 0.057
Detector resolution: 13.6612 pixels mm^-1^	θ_max_ = 27.5°, θ_min_ = 3.0°
ω scans	*h* = −10→10
Absorption correction: multi-scan (*CrystalClear*; Rigaku, 2005)	*k* = −17→17
*T*_min_ = 0.491, *T*_max_ = 0.571	*l* = −15→15
13757 measured reflections	

### Refinement

**Table:**

Refinement on *F*^2^	Primary atom site location: structure-invariant direct methods
Least-squares matrix: full	Secondary atom site location: difference Fourier map
*R*[*F*^2^ > 2σ(*F*^2^)] = 0.046	Hydrogen site location: inferred from neighbouring sites
*wR*(*F*^2^) = 0.135	H atoms treated by a mixture of independent and constrained refinement
*S* = 0.98	*w* = 1/[σ^2^(*F*_o_^2^) + (0.069*P*)^2^ + 4.1266*P*] where *P* = (*F*_o_^2^ + 2*F*_c_^2^)/3
3152 reflections	(Δ/σ)_max_ = 0.001
149 parameters	Δρ_max_ = 0.58 e Å^−^^3^
0 restraints	Δρ_min_ = −0.52 e Å^−^^3^

### Special details

**Table:**

Geometry. All e.s.d.'s (except the e.s.d. in the dihedral angle between two l.s. planes) are estimated using the full covariance matrix. The cell e.s.d.'s are taken into account individually in the estimation of e.s.d.'s in distances, angles and torsion angles; correlations between e.s.d.'s in cell parameters are only used when they are defined by crystal symmetry. An approximate (isotropic) treatment of cell e.s.d.'s is used for estimating e.s.d.'s involving l.s. planes.
Refinement. Refinement of *F*^2^ against ALL reflections. The weighted *R*-factor *wR* and goodness of fit *S* are based on *F*^2^, conventional *R*-factors *R* are based on *F*, with *F* set to zero for negative *F*^2^. The threshold expression of *F*^2^ > σ(*F*^2^) is used only for calculating *R*-factors(gt) *etc*. and is not relevant to the choice of reflections for refinement. *R*-factors based on *F*^2^ are statistically about twice as large as those based on *F*, and *R*- factors based on ALL data will be even larger.

### Fractional atomic coordinates and isotropic or equivalent isotropic displacement parameters (Å^2^)

**Table:**

	*x*	*y*	*z*	*U*_iso_*/*U*_eq_	
Co1	0.22754 (6)	1.23132 (4)	−0.01115 (4)	0.02235 (17)	
Cl2	0.22305 (12)	1.24111 (8)	−0.19820 (7)	0.0304 (2)	
Cl3	0.19972 (12)	1.39179 (7)	0.04142 (8)	0.0276 (2)	
Cl4	0.00899 (12)	1.14672 (8)	0.04230 (8)	0.0321 (2)	
Cl1	0.46675 (12)	1.16243 (8)	0.04912 (8)	0.0325 (2)	
N2	0.1021 (4)	0.8570 (2)	0.3083 (3)	0.0232 (7)	
C8	0.5802 (5)	1.0508 (3)	0.2980 (4)	0.0295 (9)	
N1	0.3699 (3)	0.9263 (2)	0.2626 (2)	0.0179 (6)	
C7	0.5319 (5)	0.9636 (3)	0.2328 (3)	0.0254 (8)	
H7A	0.6120	0.9123	0.2458	0.031*	
H7B	0.5270	0.9801	0.1552	0.031*	
C2	0.3635 (5)	0.9171 (4)	0.3857 (3)	0.0304 (9)	
H2A	0.3699	0.9817	0.4192	0.037*	
H2B	0.4545	0.8786	0.4154	0.037*	
C6	0.0766 (5)	0.9545 (3)	0.2549 (4)	0.0328 (9)	
H6A	0.0331	1.0004	0.3062	0.039*	
H6B	0.0000	0.9485	0.1916	0.039*	
C5	0.1811 (5)	0.7878 (3)	0.2331 (3)	0.0284 (9)	
H5A	0.1173	0.7831	0.1637	0.034*	
H5B	0.1883	0.7228	0.2657	0.034*	
C4	0.2072 (5)	0.8675 (3)	0.4119 (3)	0.0275 (8)	
H4A	0.2297	0.8033	0.4439	0.033*	
H4B	0.1526	0.9066	0.4646	0.033*	
C1	0.2367 (5)	0.9922 (4)	0.2188 (4)	0.0395 (11)	
H1A	0.2344	0.9939	0.1391	0.047*	
H1B	0.2553	1.0585	0.2460	0.047*	
N3	0.6229 (5)	1.1147 (3)	0.3508 (3)	0.0417 (10)	
C3	0.3479 (5)	0.8252 (3)	0.2136 (4)	0.0338 (10)	
H3A	0.4283	0.7809	0.2472	0.041*	
H3B	0.3620	0.8277	0.1353	0.041*	
H10	0.009 (6)	0.840 (4)	0.329 (4)	0.032 (12)*	

### Atomic displacement parameters (Å^2^)

**Table:**

	*U*^11^	*U*^22^	*U*^33^	*U*^12^	*U*^13^	*U*^23^
Co1	0.0229 (3)	0.0220 (3)	0.0222 (3)	0.0000 (2)	0.00147 (19)	−0.00076 (19)
Cl2	0.0287 (5)	0.0414 (6)	0.0211 (4)	−0.0016 (4)	0.0011 (3)	−0.0027 (4)
Cl3	0.0320 (5)	0.0209 (5)	0.0305 (5)	−0.0024 (4)	0.0066 (4)	−0.0011 (4)
Cl4	0.0302 (5)	0.0298 (5)	0.0366 (5)	−0.0057 (4)	0.0045 (4)	0.0038 (4)
Cl1	0.0283 (5)	0.0377 (6)	0.0312 (5)	0.0063 (4)	0.0012 (4)	0.0073 (4)
N2	0.0190 (15)	0.0256 (17)	0.0257 (15)	−0.0021 (13)	0.0063 (12)	−0.0035 (13)
C8	0.027 (2)	0.023 (2)	0.038 (2)	−0.0038 (17)	−0.0002 (16)	0.0084 (17)
N1	0.0182 (14)	0.0165 (15)	0.0190 (14)	−0.0004 (12)	0.0023 (11)	−0.0008 (11)
C7	0.0220 (18)	0.026 (2)	0.0294 (19)	−0.0053 (15)	0.0074 (15)	0.0016 (15)
C2	0.0255 (19)	0.047 (3)	0.0185 (17)	−0.0080 (18)	0.0007 (14)	−0.0004 (17)
C6	0.025 (2)	0.031 (2)	0.043 (2)	0.0093 (17)	0.0031 (17)	0.0025 (18)
C5	0.0262 (19)	0.026 (2)	0.034 (2)	−0.0063 (16)	0.0053 (16)	−0.0129 (16)
C4	0.030 (2)	0.035 (2)	0.0182 (16)	−0.0063 (17)	0.0057 (15)	−0.0023 (15)
C1	0.028 (2)	0.031 (2)	0.059 (3)	0.0022 (18)	−0.005 (2)	0.020 (2)
N3	0.052 (2)	0.028 (2)	0.044 (2)	−0.0136 (18)	−0.0076 (18)	0.0070 (17)
C3	0.034 (2)	0.026 (2)	0.044 (2)	−0.0080 (17)	0.0192 (19)	−0.0181 (18)

### Geometric parameters (Å, º)

**Table:**

Co1—Cl1	2.2749 (12)	C2—C4	1.516 (5)
Co1—Cl4	2.2808 (12)	C2—H2A	0.9700
Co1—Cl2	2.2809 (11)	C2—H2B	0.9700
Co1—Cl3	2.2910 (12)	C6—C1	1.518 (6)
N2—C6	1.487 (5)	C6—H6A	0.9700
N2—C4	1.493 (5)	C6—H6B	0.9700
N2—C5	1.496 (5)	C5—C3	1.510 (6)
N2—H10	0.86 (5)	C5—H5A	0.9700
C8—N3	1.125 (6)	C5—H5B	0.9700
C8—C7	1.469 (6)	C4—H4A	0.9700
N1—C1	1.495 (5)	C4—H4B	0.9700
N1—C7	1.505 (4)	C1—H1A	0.9700
N1—C3	1.506 (5)	C1—H1B	0.9700
N1—C2	1.510 (5)	C3—H3A	0.9700
C7—H7A	0.9700	C3—H3B	0.9700
C7—H7B	0.9700		
			
Cl1—Co1—Cl4	113.26 (5)	N2—C6—C1	109.0 (3)
Cl1—Co1—Cl2	107.64 (5)	N2—C6—H6A	109.9
Cl4—Co1—Cl2	110.73 (5)	C1—C6—H6A	109.9
Cl1—Co1—Cl3	113.85 (5)	N2—C6—H6B	109.9
Cl4—Co1—Cl3	107.70 (4)	C1—C6—H6B	109.9
Cl2—Co1—Cl3	103.21 (4)	H6A—C6—H6B	108.3
C6—N2—C4	110.1 (3)	N2—C5—C3	109.2 (3)
C6—N2—C5	110.4 (3)	N2—C5—H5A	109.8
C4—N2—C5	108.9 (3)	C3—C5—H5A	109.8
C6—N2—H10	105 (3)	N2—C5—H5B	109.8
C4—N2—H10	106 (3)	C3—C5—H5B	109.8
C5—N2—H10	116 (3)	H5A—C5—H5B	108.3
N3—C8—C7	176.4 (5)	N2—C4—C2	109.0 (3)
C1—N1—C7	111.4 (3)	N2—C4—H4A	109.9
C1—N1—C3	109.8 (3)	C2—C4—H4A	109.9
C7—N1—C3	107.4 (3)	N2—C4—H4B	109.9
C1—N1—C2	109.3 (3)	C2—C4—H4B	109.9
C7—N1—C2	111.0 (3)	H4A—C4—H4B	108.3
C3—N1—C2	107.8 (3)	N1—C1—C6	109.7 (3)
C8—C7—N1	111.0 (3)	N1—C1—H1A	109.7
C8—C7—H7A	109.4	C6—C1—H1A	109.7
N1—C7—H7A	109.4	N1—C1—H1B	109.7
C8—C7—H7B	109.4	C6—C1—H1B	109.7
N1—C7—H7B	109.4	H1A—C1—H1B	108.2
H7A—C7—H7B	108.0	N1—C3—C5	109.5 (3)
N1—C2—C4	109.5 (3)	N1—C3—H3A	109.8
N1—C2—H2A	109.8	C5—C3—H3A	109.8
C4—C2—H2A	109.8	N1—C3—H3B	109.8
N1—C2—H2B	109.8	C5—C3—H3B	109.8
C4—C2—H2B	109.8	H3A—C3—H3B	108.2
H2A—C2—H2B	108.2		

### Hydrogen-bond geometry (Å, º)

**Table:**

*D*—H···*A*	*D*—H	H···*A*	*D*···*A*	*D*—H···*A*
N2—H10···Cl3^i^	0.86 (5)	2.52 (5)	3.236 (3)	140 (4)
N2—H10···Cl2^ii^	0.86 (5)	2.65 (5)	3.225 (3)	125 (4)
C3—H3*B*···Cl1^iii^	0.97	2.74	3.647 (4)	156
C7—H7*A*···Cl2^iii^	0.97	2.58	3.492 (4)	156
C2—H2*A*···Cl3^iv^	0.97	2.73	3.543 (4)	142
C3—H3*A*···N3^v^	0.97	2.58	2.983 (4)	105

Symmetry codes: (i) −*x*, *y*−1/2, −*z*+1/2; (ii) −*x*, −*y*+2, −*z*; (iii) −*x*+1, −*y*+2, −*z*; (iv) *x*, −*y*+5/2, *z*+1/2; (v) −*x*+1, *y*−1/2, −*z*+1/2.
